# Evolutionary Dynamics of the *CBL-CIPK* Gene Families in Five Grasses and Expression/Interaction Analysis in Rice: Focus on an *OsCBL4*-Associated Module

**DOI:** 10.3390/genes17030345

**Published:** 2026-03-19

**Authors:** Mengting Huang, Siyuan Huang, Yinhua Chen, Yanke Lu, Xiaowei Yan, Yong Yun, Funeng Xing, Qingjie Tang, Xiaorong Xiao

**Affiliations:** 1 State Key Laboratory of Tropical Crop Breeding, Sanya Institute of Breeding and Multiplication, Hainan University, Sanya 570025, China; 23210901000039@hainanu.edu.cn (M.H.); 21110901000011@hainanu.edu.cn (S.H.); yhchen@hainanu.edu.cn (Y.C.); 2 Cereal Crops Institute, Hainan Academy of Agricultural Sciences, Haikou 571100, China; yanxiaowei@hnaas.org.cn (X.Y.); yunyong3819@163.com (Y.Y.); xfn6653@163.com (F.X.); 3 Sanya Institute, Hainan Academy of Agricultural Sciences, Sanya 572025, China; 4 Hubei Key Laboratory of Biological Resources Protection and Utilization, Hubei Minzu University, Enshi 445000, China; luyanke2009@126.com

**Keywords:** rice, OsCBL-OsCIPK module, gene family, gene expression, biotic stress, abiotic stress

## Abstract

Background: The Calcineurin B-like (CBL) and CBL-interacting protein kinase (CIPK) system constitute critical signaling modules mediating plant responses to abiotic stress. Although these families have been studied across various species, their evolutionary dynamics across grasses and the functional plasticity of specific isoforms remain elusive. Methods: A genome-wide analysis of CBL and CIPK families was conducted across five major Poaceae species (*Oryza sativa*, *Triticum aestivum*, *Zea mays*, *Sorghum bicolor*, and *Saccharum spontaneum*). Phylogenetic and synteny analyses were analyzed to family expansion and evolution. Cis-regulatory elements analysis in gene promoter regions were examined to predict potential stress-responsive features. Expression profiles of *OsCBL* and *OsCIPK* gene families were examined by qRT-PCR under conditions involving PEG-induced osmotic stress, pathogen strain P6 inoculation, and exogenous application of the phytohormones abscisic acid (ABA) and methyl jasmonate (MeJA). Protein–protein interactions between selected CBL (OsCBL4) and CIPK pairs were assessed via Yeast Two-Hybrid (Y2H) and Luciferase Complementation Imaging assays (LCI). Results: Phylogenetic and synteny analyses indicated that segmental duplications have contributed substantially to the expansion of these gene families. Promoter analysis revealed that the majority of *CBL* and *CIPK* family members, exemplified by *OsCBL4*, traditionally characterized as a salt sensor, possesses a cis-element architecture (rich in ABREs and MBS) heavily biased towards dehydration responsiveness. Expression profiling showed that *OsCBL4* is significantly hyper-induced by direct osmotic stress (PEG) but exhibits almost no response to exogenous ABA. A subset of kinases genes (e.g., *OsCIPK2*, *9*, *18*) displayed PEG-induced expression patterns resembling those of *OsCBL4*, whereas *OsCIPK30* remained transcriptionally unresponsive under the same conditions. Protein interaction assays demonstrated that OsCBL4 physically interacts exclusively with PEG-responsive transcriptionally activated kinases such as OsCIPK9, but failed to interact with the non-responsive OsCIPK30. Conclusions: Our study provides a genomic characterization of *CBL* and *CIPK* families across five major Poaceae species. The combined expression and interaction data reveal that *OsCBL4*-assembles with specific *CIPKs* into signaling modules during osmotic stress responses in rice, pointing to roles that go beyond salt stress responses. The findings establish a foundation for further functional dissection of CBL-CIPK pathway diversification in abiotic stress adaptation.

## 1. Introduction

Plants have established a series of defense mechanisms to adapt to the biotic and abiotic stresses during their entire lifecycle [1]. Elucidating the underlying signaling pathways is essential for enhancing plant resilience and crop productivity. Among these, the calcium-sensing CBL-CIPK network plays a pivotal role [2]. Under stress conditions, CBL proteins serve as Ca^2+^ sensors, perceiving Ca^2+^ signals via four conserved EF-hand (elongation factor-hand) motifs [3]. Upon binding Ca^2+^, the CBL proteins interacted with their downstream target proteins kinase CIPK through the C-terminal NAF domain of CIPK [4], thereby forming functional CBL-CIPK complexes that relay stress signals [2].

The functional versatility of this network is well-documented. The *AtCBL4-AtCIPK24* (also known as *SOS2-SOS3*) module was first uncovered in *Arabidopsis thaliana* for its role in salinity tolerance [5]. Subsequent research revealed tissue-specific variations, such as AtCBL10-AtCIPK24 module regulating Na^+^ homeostasis in vacuolar membrane [6], and alternative complexes like AtCBL10-AtCIPK8 also contributing to salt stress response [7,8]. The SOS pathway was conserved in various plant species such as rice, mustard, apple and poplar [9,10]. Beyond salinity, the network also mediates abscisic acid-dependent and independent drought stress responses via alternative CBL-CIPK pairings, as seen with AtCIPK1 [11]. Additionally, recent evidence implicates these CBL-CIPK modules in plant immune signing, with *SiCBL10-SiCIPK6* module integrating Ca^2+^ and ROS signaling during pathogen defense [12,13]. These studies highlight the complexity of the CBL-CIPK network, wherein one CBL can target multiple CIPKs, and conversely, a single CIPK can interact with different CBLs [14].

Approximately 10 CBLs and 33 CIPKs have been identified through genome-wide analyses in rice [15,16]. Despite this characterization, functional studies have largely been limited to expression patterns analysis of individual genes. Only a few modules have been functionally validated, including OsCBL8–OsCIPK17 involved in multiple stress responses and OsSOS3-OsCIPK9 mediating salt tolerance [17,18]. To date, no systematic analysis has been conducted on the interaction networks and evolutionary dynamics of CBL-CIPK modules in grasses, leaving these critical aspects poorly understood.

In this study, we performed phylogenetic analyses of CBL-CIPK families in grasses. We examined collinearity and repetitive sequences, subcellular localization prediction, cis-acting elements, expression dynamics in response to multiple stresses and protein–protein interaction networks of the rice CBL and CIPK families. Through Yeast Two-Hybrid and luciferase complementation experiments, we identified potential CBL-CIPK modules. Our work provides a new perspective on the evolutionary divergence of these gene families and establishes a foundation for deciphering the role of the OsCBL4-mediated signaling network in multiple stress resistance.

## 2. Materials and Methods

### 2.1. Phylogenetic Analysis of CBL and CIPK Families in Five Grass Crops

Phylogenetic analysis of *CBL* and *CIPK* families of Gramineae obtained genomic data of rice, sorghum and maize through phytozome database (https://phytozome-next.jgi.doe.gov/, accessed on 21 March 2025), and genomic data of wheat and sugarcane from phytozome database EnsemblPlants database (https://plants.ensembl.org/, accessed on 21 March 2025). The protein sequences of *CBL* and *CIPK* genes of five species of Gramineae were extracted by TBtools v2.423. The protein amino acid sequences of Gramineae CBL and CIPK were compared by MEGA12 software, and the subfamilies of Gramineae CBL and CIPK were classified. The phylogenetic tree of CBL and CIPK of Gramineae was constructed by MEGA12 software neighbor merging method (Neighbor-joining method), and the phylogenetic tree was beautified by the online website TVbot (https://chiplot.online/tvbot.html, accessed on 8 December 2025).

### 2.2. Identification of Promoter Cis-Regulatory Elements

To identify the cis-elements in the promoter, we extract the upstream 2000 bp regions of two gene families using TBtools software. Then, the obtained sequence files were submitted to PlantCARE website (https://bioinformatics.psb.ugent.be/webtools/plantcare/html/ (accessed on 20 June 2025)). After filtering the obtained result files, classify them according to the function of cis-acting elements into five categories: light response elements, growth and development respond elements, biotic/abiotic stress respond elements, phytohormone respond elements, and MYB response elements, and perform visual analysis of the predicted elements using TBtools-II.

### 2.3. Gene Duplication Analysis of the OsCBL and OsCIPK Gene Families

Using the rice genome annotation file, we conducted a collinearity analysis of the *OsCBL* and *OsCIPK* genes with the aid of TBtools software. Additionally, we employed TBtools to perform a visual analysis of the chromosome distribution of *OsCBL* and *OsCIPK* in rice, as well as to examine the gene collinearity merger.

### 2.4. Co-Expression Network and Subcellular Localization Prediction Analysis

The obtained protein sequences were used to predict the subcellular localization of rice OsCBL and OsCIPK using the WoLF PSORT website (https://wolfpsort.hgc.jp/, accessed on 7 January 2026), and the prediction results were visualized using TBtools software. The STRING database (https://cn.string-db.org/, accessed on 22 December 2025) was used to predict the interaction networks of the rice OsCBL and OsCIPK gene family protein sequences, and the obtained prediction results were analyzed and beautified using Cytoscape v3.10.4 software.

### 2.5. Stress Treatments

Rice seeds (*O. sativa* L. ssp. *japonica* cv Nipponbare) were germinated, and seedings were grown hydroponically in Hoagland solution under controlled conditions (14 h light/10 h dark cycle). Upon reaching the three-leaf-one-heart stage, the nutrient solution was replaced with sterile distilled water for a 24 h adaptation period prior to stress treatments. For stress treatments, plants were inoculated with *Xoo* strain P6 (OD600 = 0.5) using the leaf-clip method, exposed to 20% PEG6000 solution for osmotic stress, or sprayed with 150 µM ABA or 100 µM MeJA, respectively. Leaf and leaf sheath samples (1 g total fresh weight) were collected at 0 and 24 h post-treatment (hpt). Ten independent samples were taken at each time point, constituting pooled biological replicates. The collected samples were immediately flash-frozen in liquid nitrogen for 10 min and then stored at −80 °C for subsequent expression analysis.

### 2.6. RNA Extraction and qRT-PCR Analysis

Leaf and leaf sheath samples from stressed rice plants were thoroughly ground in liquid nitrogen. Approximately 0.1 g of the ground tissue was transferred to RNase-free sterile microcentrifuge tubes, and total RNA was extracted using 1 mL of TRNzol Unicersal reagent (DP424, TIANGEN Biotech, Beijing, China) following the manufacturer’s protocol. Genomic DNA contamination was eliminated by treating the RNA samples with RNase-free DNase I (D2215, TaKaRa, Tokyo, Japan). First-strand cDNA synthesis was performed with 1 μg of total RNA using a reverse transcription kit (TCH026, TaKaRa, Japan). The resulting cDNA was diluted appropriately and used as the template for qRT-PCR analysis.

Gene-specific primers for qRT-PCR were designed with the assistance of Primer 5 software. qRT-PCR was carried out on a real-time PCR system using TB Green Premix Ex Taq II (RR820B, TaKaRa, Japan). The thermal cycling conditions were as follows: initial denaturation at 95 °C for 30 s, followed by 40 cycles of 95 °C for 5 s and 60 °C for 30 s. A melting curve analysis was performed at the end of each run to verify amplification specificity. The rice *OsUBQ10* gene was used as the internal reference for normalization. Relative gene expression levels were calculated using the 2^−ΔΔCt^ method. All experiments were performed with pooled biological replicates and three technical replicates. The sequences of all primers used in this study are presented in Table S1.

### 2.7. Luciferase Complementation Assay

Constructed the OsCBL-NAU-PMG ab prtcl-24.3-pC1300-nLUC vector and the OsCIPK-NAU-PMG Lab prtcl-24.3-pC1300-cLUC vector (Primer sequences are listed in Table S2). Using the *Nicotiana benthamiana* transient expression system, the plant expression vectors containing the fusion proteins were transformed into Agrobacterium tumefaciens and then injected into tobacco leaves. After 24 h of darkness and 24 h of light, luciferase substrate was added, and fluorescence intensity was qualitatively detected using a plant in vivo molecular imaging system (CCD imaging system) or luminometer to determine whether there is an interaction between the target proteins and the degree of their interaction.

### 2.8. Yeast Two-Hybrid (Y2H) Experiment

Construct the OsOsCBL-pGADT7 vector and OsCIPK-pGBKT7 vector (Primer sequences are listed in Table S2), using PGBDT7-Lam/PGADT7-LargeT as the negative control and PGBDT7-53/PGADT7-LargeT as the positive control. Using a Y2H system, co-transform the target genes into yeast Y2HGold. Spot the positive clone yeast on SD/-TL and SD/-TLHA selective media and incubate for 2–4 days, then observe their growth.

### 2.9. Statistical Analysis

All qRT-PCR data were generated from at least three independent replicates and expressed as mean ± standard error of the mean (SEM). To identify genes with significant expression changes between 0 h and 24 h post-treatment, multiple *t*-tests (unpaired *t*-tests with Holm–Šídák correction) method was employed. Additionally, two-way analysis of variance (ANOVA) was utilized to assess the main effects of genotype, treatment, and their interaction. Statistical significance was defined as *p* < 0.05. All statistical analyses were performed using R software (version 4.4.2).

## 3. Results

### 3.1. Phylogenetic Analysis Reveals Significant Expansion of CBL and CIPK Families in Five Grass Species

**Figure 1:**
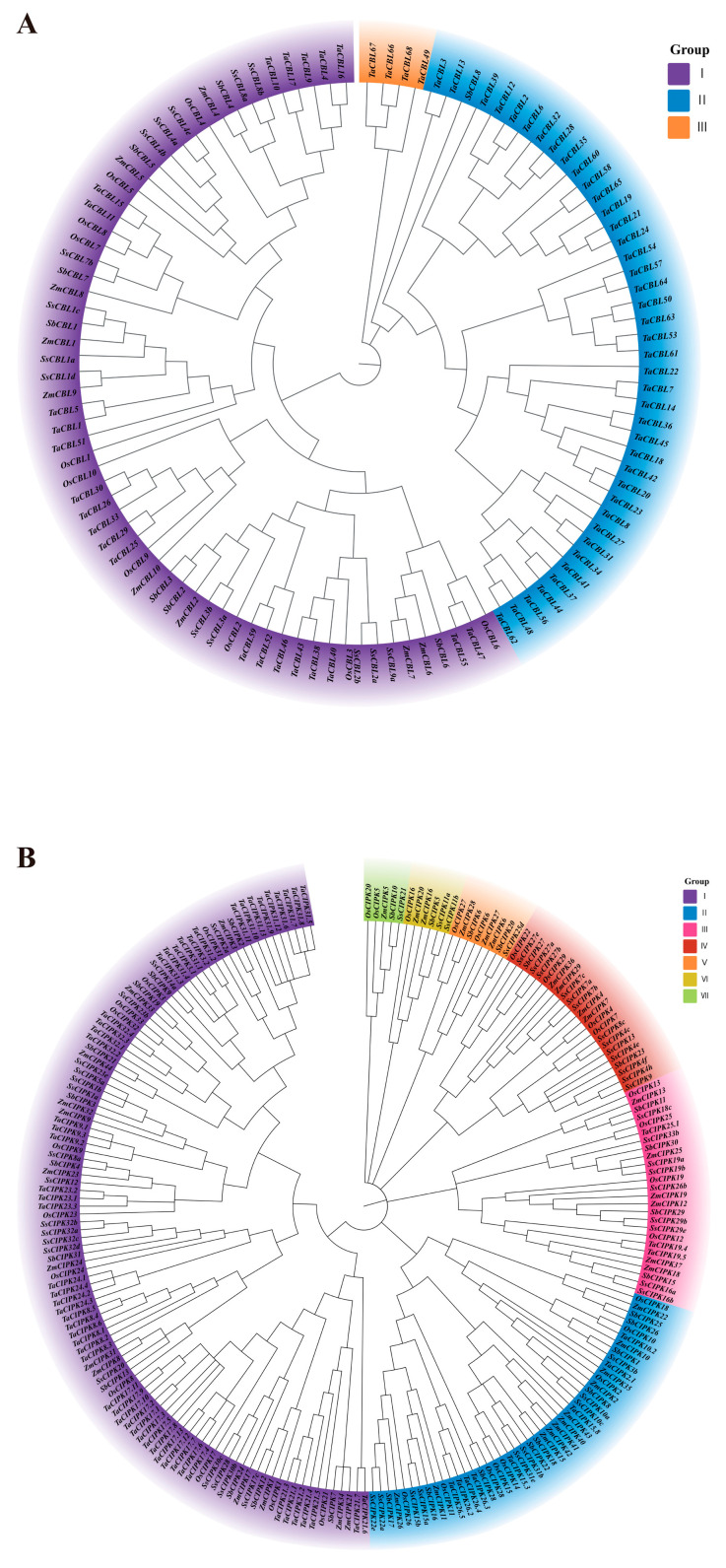
The phylogenetic tree of the CBL and CIPK families in grasses was constructed using sequences from rice (*O. sativa*), wheat (*T. aestivum*), maize (*Z. mays*), sorghum (*S. bicolor*), and sugarcane (*Saccharum*). This phylogenetic tree was built using the Neighbor-joining method with 1000 bootstrap replicates. The tree includes colored blocks to represent different evolutionary branches. I–VII represent different clusters. (**A**) CBL protein families. (**B**) CIPK protein families.

The phylogenetic relationships of the CBL and CIPK families across five gramineous crops—rice (*O. sativa*), wheat (*T. aestivum*), corn (*Z. mays*), sorghum (*S. bicolor*) and sugarcane (*S. spontaneum*)—were analyzed (Figure 1 and Figure S1). The topological structures of the coding region and amino acid sequence trees are largely consistent, which indicates that the CBL and CIPK families have experienced relatively conserved selective pressure during Poaceae evolution. In the tree diagram, we can clearly see that “Os-Zm-Sb-Ss” are often clustered together. For instance, *OsCBL1*, *ZmCBL1* and *SbCBL1* coalesce into one. This means that these genes already existed before the differentiation of the ancestors of the Poaceae family.

The CBL family acts as an early sensor in calcium-mediated stress signaling. The CBL family has a relatively small number of members (usually 10 for rice) and is divided into three main subfamilies (Group I, II, III) (Figure 1A). Group I (the branch where *OsCBL1*/*OsCBL9* is located) is usually extremely conservative in the plant kingdom. The evolutionary tree shows that *OsCBL1* is highly similar to the homologous genes of sorghum and corn. We observed the clustering of *OsCBL4*, *ZmCBL4* and *SbCBL4*. This is a member of the classic SOS (Salt Overly Sensitive) pathway. Group III has the fewest members, and rice contains only a small amount of OsCBL members.

CIPK act as kinases and are responsible for converting calcium signals into phosphorylation events. The results showed that the family was divided into seven groups (Group I–VII) (Figure 1B), mainly into two major categories: intron-rich and intron-low. The number of genes in wheat (Ta) and sugarcane (Ss) is much greater than that in rice, consistent with their polyploid genomes. Rice, as a diploid model plant, has the most concise genome. In wheat, a certain CIPK may have three copies (*TaCIPK23.1*, *23.2*, *23.3*), while in rice, there is usually only one (*OsCIPK23*). The lower functional redundancy in the diploid rice genome makes it a highly suitable model for baseline functional characterization. In the tree graph, *OsCIPK23* is clustered with *ZmCIPK23*, while *OsCIPK24* is clustered with *ZmCIPK24*.

This phylogenetic tree not only demonstrates the kinship among species but also suggest potential functional conservation related to abiotic stress responses, though in planta functional validation remains necessary.

### 3.2. Synteny and Duplication Analysis Highlight the Evolutionary Dynamics of the OsCBL-OsCIPK Network

**Figure 2:**
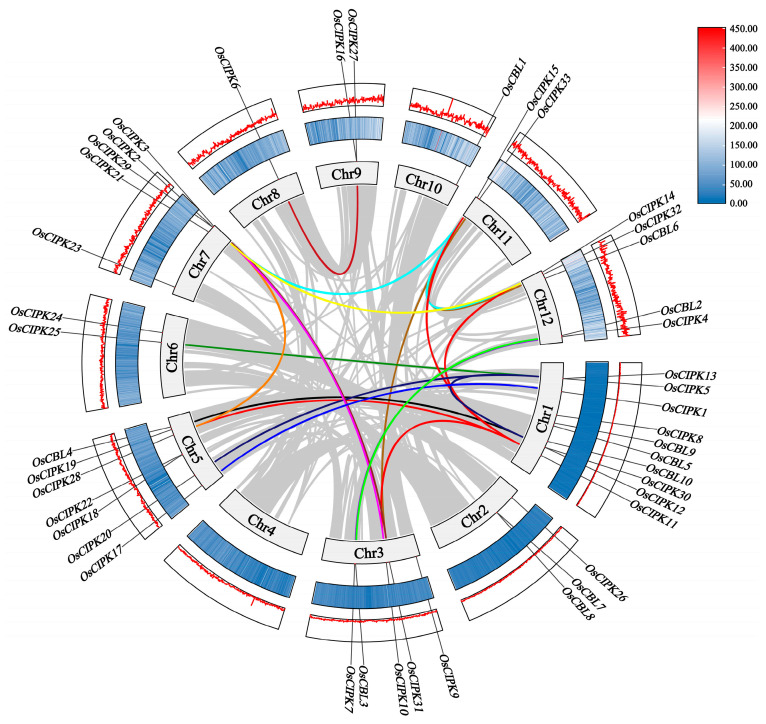
Analysis of chromosome location and collinearity of *OsCBL-OsCIPK.* The chromosome location information of *OsCBL-OsCIPK* genes and their intraspecific gene duplication collinearity analysis. Different colored lines connecting interchromosomal regions represent *OsCBL-OsCIPK* gene pairs, while gray lines represent syntenic gene pairs in the background of the rice genome.

Collinearity and duplication analyses were performed to investigate the evolutionary expansion of these gene families. This picture shows the positional relationship and homology of *OsCBL* and *OsCIPK* genes on the 12 rice chromosomes (Chr1-Chr12). We can see that *OsCBL* (10) and *OsCIPK* (33) genes are not evenly distributed (Figure 2). Chr1 gathers a large number of *OsCIPK* genes (such as *OsCIPK1*, *5*, *8*, *9*, *10*, *11*, *12*, *13*, *30*). In addition, Chr3 and Chr7 are also more widely distributed. Some chromosomes such as Chr10 and Chr8 are less distributed. Combining the red gene density curve and blue-white heat map in the figure, the vast majority of CBL/CIPK genes are located in euchromatin regions with high gene density and active transcription, avoiding the heterochromatin regions near centromeres.

The dense cross-chromosome connections in the figure (such as the lines connecting Chr1 and Chr5, Chr2 and Chr6) represent paralogous pairs (Figure 2). It shows that segmental duplication is the main driving force for the expansion of CBL and CIPK families in rice. This is highly consistent with the Whole Genome Duplication (WGD) event that occurred in the evolutionary history of rice. The dense CIPK cluster on Chr1 suggests that local tandem duplication events may have occurred here, resulting in the clustering of genes.

It can be seen from the figure that CIPK has far more connections and labels than CBL (Figure 2). This evolutionary expansion indicates that rice has developed a duplicated and potentially redundant CBL-CIPK calcium signaling network allowing a limited number of OsCBL sensors to theoretically interact with diverse downstream OsCIPKs.

### 3.3. Structural Features and Promoter Analysis Suggest Potential Roles in Drought Stress Responses

As two core families in the calcium-dependent protein kinase network, the 10 members of the OsCBL family and the 33 members of the OsCIPK family are predicted to be distributed in chloroplasts (chlo), cytoplasm (cyto), nuclei (nucl), mitochondria (mito), and plasma membranes (plas), etc. (Figure S2). Based on prediction scores, OsCBL proteins can be categorized into three major groups (Figure S2A): The first group shows high scores in both cytoplasmic and nuclear localization subpopulations (OsCBL1, OsCBL2, OsCBL3, OsCBL6, OsCBL7). The scores of OsCBL1 and OsCBL2 at these two positions are balanced and relatively high. The second group exhibits high confidence scores for chloroplast localization (OsCBL4, OsCBL8, OsCBL9, OsCBL10), with OsCBL4 reaching a score of 13.00. OsCBL5 is rather unique. It has the highest (score 6.00) at the ‘mito’ (mitochondria) and also has cytoplasmic localization, which may be involved in regulating the calcium ion balance of mitochondria. Although the predicted scores were not as high as those of chloroplasts, OsCBL9 and 10 showed significant scores (3.00/2.00) at the ‘plas’ (plasma membrane). They may be involved in regulating the ion channels and osmotic pressure of guard cells.

A notable feature is that the vast majority of OsCIPK members have extremely high predicted scores in chloroplasts (chlo) (such as OsCIPK12, 19, 21, and 32, with scores of 10–14) (Figure S2B). This suggests that the CIPK family of rice has retained a strong ability to regulate chloroplast function in evolution. Some members (such as OsCIPK10, 11, 14, and 26) show a distinct nuclear localization tendency, which is usually related to gene expression regulation. A few OsCIPKs display specific compartment signals, including OsCIPK29 at the plasma membrane (score: 8.00), OsCIPK23 in the mitochondria and OsCIPK20 in the endoplasmic reticulum (E.R.). The bioinformatic prediction shows diverse spatial overlaps between specific OsCBLs and OsCIPKs across structural compartments (Figure S2).

**Figure 3:**
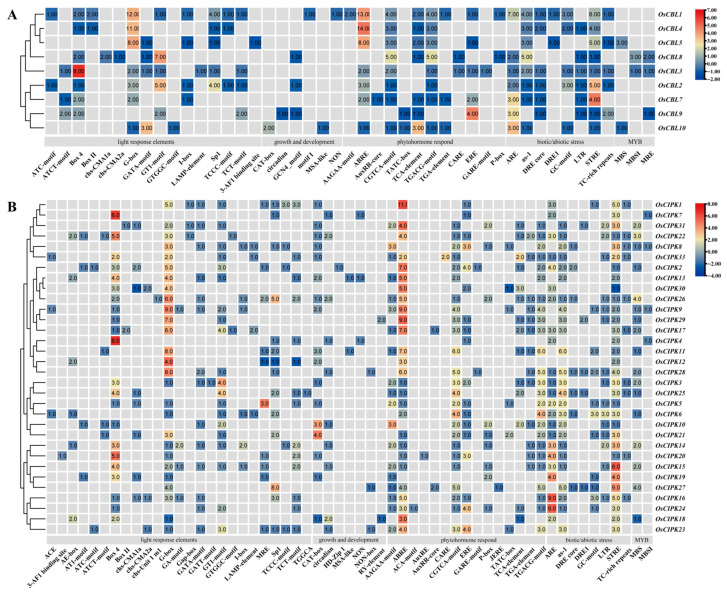
Analysis of cis-acting elements in the promoter of the *OsCBL* and *OsCIPK* families. (**A**) *OsCBL* families. (**B**) *OsCIPK* families. Five categories of cis-acting elements are shown: light response elements, growth and development, phytohormone respond, biotic/abiotic stress, and MYB stress-responsive binding site elements. Values in squares represent element counts calculated via PlantCare. The legend on the right shows scores and corresponding colors from TBtools analysis, reflecting row data in the matrix after Row Scale. In single-row data, a deeper red indicates more components, while a blue hue signifies fewer. Grey blocks denote absence of components.

The promoter region is the “control panel” for gene expression regulation. By analyzing the cis-acting elements in silico above, we can predict from the source how these genes are “turned on” or “turned off” by environmental signals (such as drought, hormones, light). Judging from the distribution of color blocks in the overall heat map, the promoter regions of the *OsCBL* and *OsCIPK* families are rich in extremely diverse cis-acting elements (Figure 3).

In drought-resistant breeding, what we are most concerned about is whether genes can respond quickly to water shortage signals. This mainly depends on hormone pathways (especially ABA) and transcription factor regulatory pathways. Specifically, ABA-responsive elements (ABREs) are highly enriched in *OsCBL4* (14 ABREs) and *OsCBL1* (13 ABREs) (Figure 3A), as well as in *OsCIPK1* (11), *OsCIPK9* (9), *OsCIPK29* (9), and *OsCIPK17* (7) (Figure 3B). Furthermore, MYB binding sites (MBS), which are typically responsive to drought induction, are present in *OsCBL1* (6 MBS) and *OsCIPK26* (4 MBS). Other stress-related motifs identified include stress response elements (STRE) and low-temperature responsiveness (LTR) motifs enriched in *OsCIPK15*, and jasmonate-responsive elements (As-1/TGACG) in *OsCIPK16*. Additionally, light-responsive elements, such as G-box and Box 4, are widely distributed, with *OsCBL4* and *OsCIPK9* containing 11 and 9 G-boxes, respectively (Figure 3).

### 3.4. Transcriptional Profiling Identifies OsCBL4 and a Specific Subset of OsCIPKs as Core Drought-Responsive Genes

**Figure 4:**
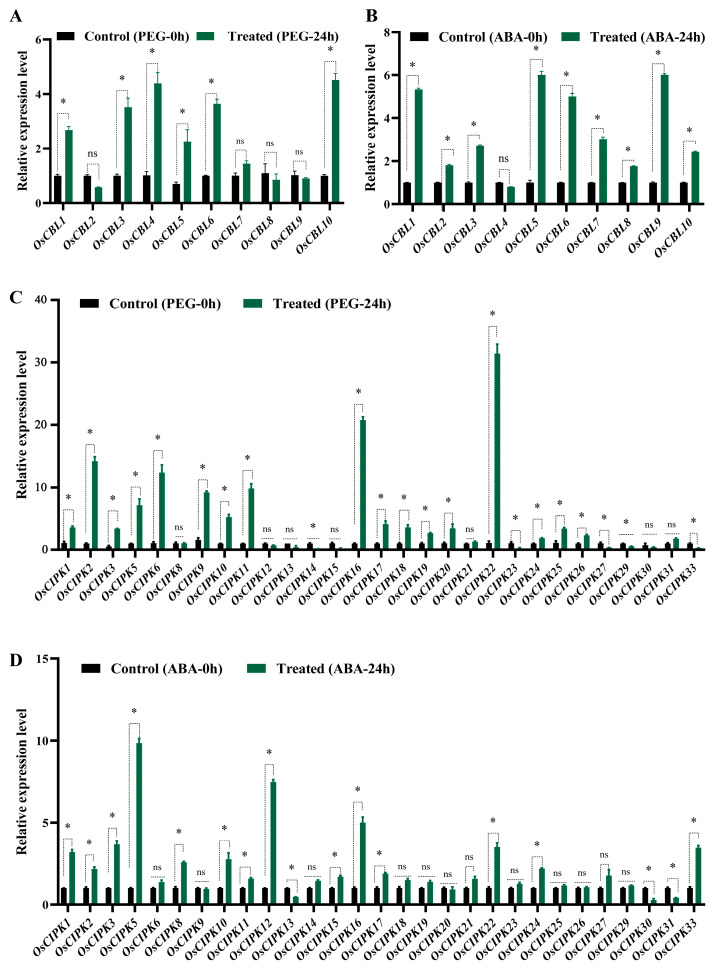
Expression patterns of *OsCBL* and *OsCIPK* families in response to PEG and AbA stresses. (**A**) Expression patterns of *OsCBL* gene families under PEG-induced osmotic stress. (**B**) Expression patterns of *OsCBL* gene families under AbA stress. (**C**) Expression patterns of *OsCIPK* gene families under PEG stress. (**D**) Expression patterns of *OsCIPK* gene families under AbA stress. Expression levels were normalized to *OsUBQ10* and are shown relative to 0 h, calculated using the 2^−∆∆Ct^ method. Data represent means (±SEM) from three technical replicates. Asterisks indicate significant differences (*p* < 0.05). “ns” indicates not significant. Time points: 0 h (pre-treatment) and 24 h (post-treatment).

We performed expression profile analysis (qRT-PCR data) of *OsCBL* and *OsCIPK* families under various stresses. Overall, the induction magnitude of *OsCIPK* genes was generally higher than that of *OsCBL* genes (Figure 4 and Figure S3).

In the PEG-24 h data, genes like *OsCIPK2*, *OsCIPK6*, *OsCIPK9*, *OsCIPK11*, *OsCIPK16*, and *OsCIPK22* showed a substantial increase after 24 h of treatment (Figure 4C), indicating their potential role as key regulators in rice response to osmotic stress. *OsCIPK5*, *OsCIPK16* and *OsCIPK22* were both noticeably upregulated under PEG and ABA treatment (Figure 4C,D). Interestingly, *OsCIPK9* was substantially upregulated under PEG, but showed no significant induction under ABA treatment (Figure 4C,D). Additionally, genes like *OsCIPK1*, *OsCIPK3*, and *OsCIPK10* displayed some degree of upregulation.

Under the treatment of P6 pathogen, the expression level of *OsCIPK10* dramatically increased, but the response under drought (PEG) conditions was far less strong (Figure 4C and Figure S3C). *OsCIPK16* and *OsCIPK22* are highly expressed under MeJA, ABA, and PEG, indicating that they may function as generalists (Hubgene) (Figure 4C,D and Figure S3D).

Among the *OsCBL* family, members such as *OsCBL1*, *OsCBL5*, and *OsCBL6* showed significant upregulation under both PEG and ABA treatment (Figure 4A,B). In contrast, *OsCBL4* exhibited a distinct expression profile: it was significantly induced by PEG stress but remained largely unresponsive to the ABA treatment under the tested conditions (Figure 4A,B). Expression pattern analysis provides us with a reference map for the screening route, which is helpful for screening major stress resistance genes in rice.

### 3.5. Interaction Network Prediction and Y2H Assays Reveal Selective Binding of OsCBL4

**Figure 5:**
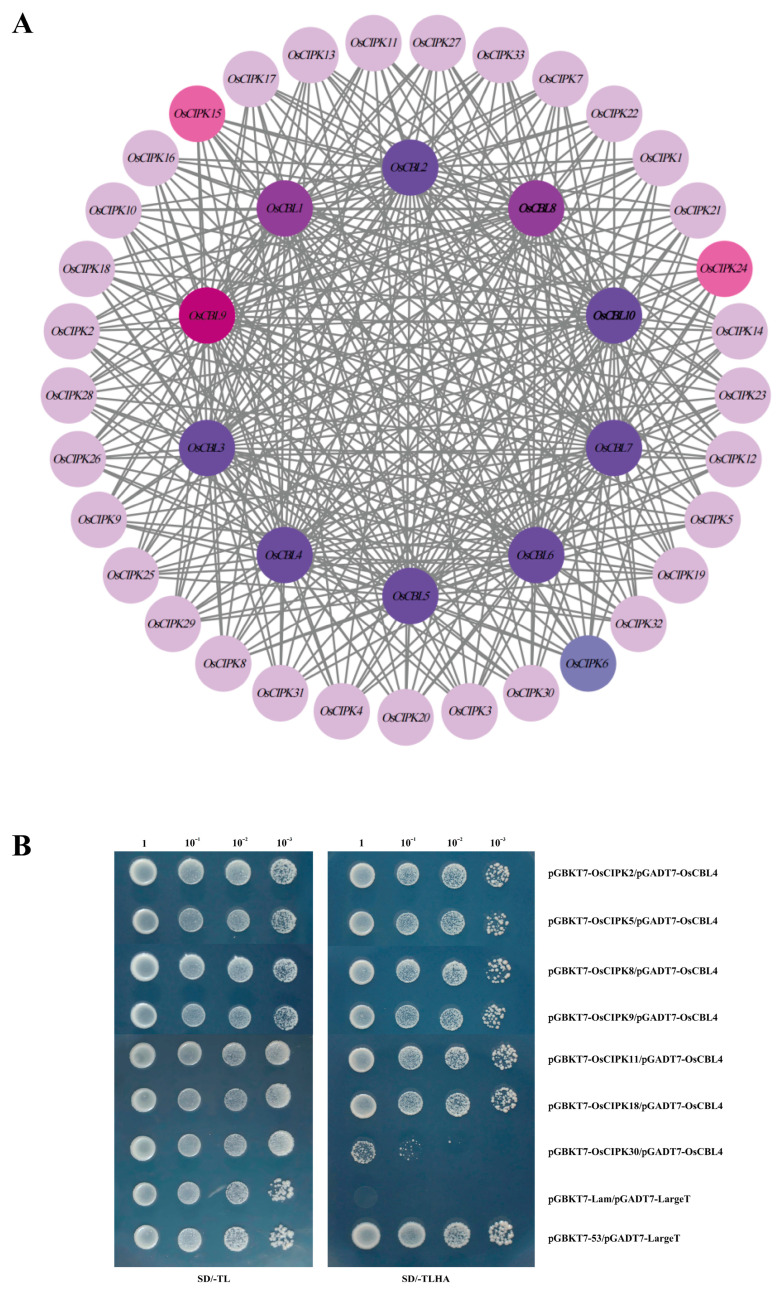
Interaction network prediction and Y2H assays reveal selective binding of OsCBL4. (**A**) Prediction of co-expression networks between OsCBL and OsCIPK. The network was generated using the STRING database. Nodes represent proteins, and gray lines indicate predicted interactions between two proteins. (**B**) Y2H assays of OsCBL4 with its candidate target proteins. Autoactivation assays for the candidate proteins are provided in Figure S4, and all showed no autoactivation. The pGBKT7-53/pGADT7-LargeT and pGBKT7-Lam/pGADT7-LargeT pairs represent positive and negative controls, respectively. In the Y2H experiment of OsCBL4, all tested proteins except OsCIPK30 showed interaction signals with OsCBL4.

The OsCBL-OsCIPK protein–protein interaction (PPI) network was predicted using the STRING database (Figure 5A). It can be intuitively observed from the figure that the dark (purple/magenta) nodes in the figure are OsCBL family members (such as OsCBL1, OsCBL2, OsCBL3, OsCBL4, OsCBL5, OsCBL9, etc.), which are located in the center of the network as core nodes. The light-colored (pink/lavender) nodes in the outer circle are OsCIPK family members (such as OsCIPK1, OsCIPK15, OsCIPK23, etc.), and they are numerous. Gray lines represent interactions between proteins. We can see that the connections are extremely dense, and almost every OsCBL has potential interactions with multiple OsCIPKs, forming a typical Many-to-Many network structure. In the figure, nodes such as OsCBL9 (darker color and prominent position) and OsCBL1 are connected to a large number of CIPKs.

Conversely, individual OsCIPKs (e.g., OsCIPK23) are predicted to connect with multiple OsCBLs (Figure 5A). Additionally, OsCBL4, conventionally associated with the SOS pathway, is predicted to interact with multiple CIPKs beyond its canonical partners (Figure 5A).

To experimentally test the interactions between OsCBL4 and several OsCIPK members that showed transcriptional responses to drought stress, we performed Y2H assays (Figure 5B). The results indicated that OsCBL4 interacted with OsCIPK2, OsCIPK5, OsCIPK8, OsCIPK9, OsCIPK11, and OsCIPK18, as evidenced by yeast growth on quadruple dropout (QDO) media, which visible signals maintained at a 10^−3^ dilution. In contrast, the combination of OsCBL4 and OsCIPK30 showed negligible growth on QDO, indicating a lack of detectable interaction under these assay conditions. Our previous expression analyses (Figure 4C,D) showed that OsCIPK2, 5, 8, 9, 11, and 18 were induced by PEG or ABA treatments, whereas OsCIPK30 exhibited low- or unresponsive expression under drought stress (Figure 4C,D).

The interaction between OsCBL4-OsCIPK2/ OsCBL4-OsCIPK18 is very strong, and the expression level of *CIPK2/18* is extremely high under PEG treatment (Figure 4C and Figure 5B). It is speculated that this pair of modules is mainly responsible for osmotic adjustment and may help rice roots retain water in water-deficient soil by phosphorylating ion channels/transporters on the tonoplast or plasma membrane. *OsCIPK5* responds strongly to ABA and interacts well with OsCBL4 (Figure 4C, Figure 5B and Figure S3C,D). It is speculated that this module is involved in the regulation of stomatal movement. *OsCIPK9* is expressed under various stress conditions and has been confirmed to interact with OsCBL4 (Figure 4C,D, Figure 5B and Figure S3C,D).

### 3.6. In Planta Validation Confirms the OsCBL4-OsCIPK9 Interaction Under Physiological Contexts

**Figure 6:**
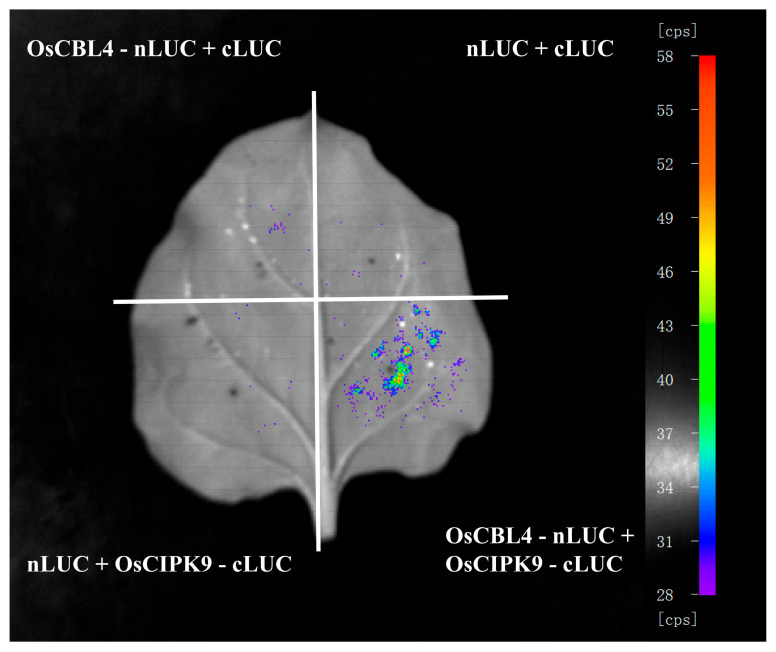
OsCBL4 interacts with OsCIPK9, as shown by split-luciferase complementation assay. Full-length *OsCBL4* and *OsCIPK9* were fused to nLUC and cLUC, respectively, and co-expressed in *N. benthamiana* leaves via agroinfiltration. Luminescence was imaged 48 h post infiltration following luciferin treatment. Color scale indicates relative luminescence intensity. Co-expression of nLUC-OsCBL4 with empty cLUC (OsCBL4 nLUC-cluc), empty nLUC with cLUC-OsCIPK9 (nLUC-OsCIPK9 cluc), and nLUC with cLUC served as negative controls. Consistent results were obtained across three biological replicates.

A firefly LCI assay was performed in tobacco (*N. benthamiana*) leaves to assess the interaction between OsCBL4 and OsCIPK9 in planta (Figure 6). The co-expression of OsCBL4-nLUC and OsCIPK9-cLUC (lower right quadrant) resulted in a distinct chemiluminescence signal, indicating the reconstitution of functional luciferase and suggesting an interaction between the two proteins in plant cells. Control combinations, including OsCBL4-nLUC with empty cLUC (upper left), empty nLUC with empty cLUC (upper right), and empty nLUC with OsCIPK9-cLUC (lower left), did not produce detectable fluorescence signals. Previous promoter analyses indicated the presence of ABRE and G-box motifs in both OsCBL4 and OsCIPK9 (Figure 3). Expression profiling showed that under the tested experimental conditions, both genes were induced by PEG treatment, but *OsCBL4* showed a minimal response to exogenous ABA application (Figure 4). The LCI results confirm that the OsCBL4 and OsCIPK9 proteins are capable of interacting in planta when co-expressed.

## 4. Discussion

### 4.1. Evolutionary Expansion and Structural Redundancy of the OsCBL-OsCIPK Network in Poaceae

The conquest of land by plants required the evolution of sophisticated signaling networks to cope with fluctuating environmental conditions, particularly a water deficit [19,20,21]. Our comparative phylogenetic analysis across five major grass species (*O. sativa*, *T. aestivum*, *Z. mays*, *S. bicolor*, and *S. spontaneum*) reveals that the CBL and CIPK gene families have undergone significant expansion, a phenomenon that is widely recognized as a key driver of adaptive evolution in plants under abiotic stress conditions [22,23,24,25]. The highly conserved topological structures observed in our phylogenetic trees suggest that the core CBL–CIPK modules were established prior to the divergence of Poaceae ancestors, underscoring their fundamental, conserved roles in calcium-mediated signal transduction. However, the varying number of family members—particularly the extensive expansion seen in polyploid crops such as wheat and sugarcane compared to diploid rice—highlights how ploidy levels and subsequent sub-functionalization contribute to lineage-specific genomic architectures.

For rice (*O. sativa*), a semi-aquatic species often cultivated in paddy fields but increasingly exposed to intermittent drought, the retention and expansion of these gene families are of particular ecological significance. Our synteny analysis demonstrates that segmental duplication has been the primary driving force behind the expansion of the OsCBL and OsCIPK families, consistent with ancient whole genome duplication (WGD) events that shaped the rice genome [26]. The retention of these duplicated genes over evolutionary time suggests that this genetic redundancy is not merely a neutral process but likely provides an evolutionary buffer, allowing plants to better tolerate genetic mutations and adapt to fluctuating environmental stresses [27].

The non-random chromosomal distribution and extensive collinearity of OsCIPK genes provide a structural basis for potential functional redundancy. In the context of abiotic stress, such structural redundancy may allow paralogous genes to compensate for one another, thereby maintaining the integrity of calcium signaling pathways under adverse conditions [28]. Moreover, the disproportion between upstream sensors (CBLs) and downstream kinases (CIPKs) supports a model of divergent signaling. This architecture implies that a limited number of calcium sensors can potentially decode calcium signatures and recruit a wider array of downstream kinases. This expanded signaling toolkit likely enables rice to finely tune its molecular responses to complex abiotic stresses, expanding its adaptive capacity without requiring a proportional increase in sensor genes [29,30,31].

### 4.2. Potential Functional Expansion of OsCBL4 in Osmotic Stress Responses

The CBL-CIPK signaling network a crucial component of plant abiotic stress tolerance. Historically, OsCBL4 (homologous to AtSOS3 in *Arabidopsis*) has been canonically characterized as a calcium sensor for the Salt Overly Sensitive (SOS) pathway [32]. In this well-established paradigm, OsSOS3/OsCBL4 perceives the calcium spike triggered by salt stress and specifically recruits OsSOS2/OsCIPK24 to activate the plasma membrane Na^+^/H^+^ antiporter SOS1, thereby maintaining ionic homeostasis [33]. While this function is critical for salinity tolerance, our integrated genomic and transcriptional analyses suggest that the regulatory scope of OsCBL4 suggest that the regulatory scope.

The in silico promoter region of *OsCBL4* is architecturally enriched with an exceptionally high density of drought-responsive cis-elements. Specifically, we identified 14 ABA-responsive elements (ABREs) and multiple MYB-binding sites (MBS)—a configuration typically characteristic of genes regulated by dehydration signals (Figure 3A) [30,34]. Paradoxically, despite this structural predisposition for ABA responsiveness, our qRT-PCR profiling showed that *OsCBL4* was significantly upregulated by PEG-simulated drought but displayed negligible responses to exogenous ABA treatment (Figure 4A,B). While this discrepancy might initially suggest an “ABA-independent” signaling pathway, this interpretation must be approached with caution. The apparent lack of response to exogenous ABA in our assays could be largely attributed to experimental factors such as “stress window effects” or tissue specificity. It is highly plausible that ABA-mediated regulation of *OsCBL4* occurs at different temporal phases or in specific tissues not fully captured by whole-seedling analysis. Furthermore, complex regulation might occur upstream or downstream of the transcript level.

Nevertheless, the strong co-induction of *OsCBL4* and a specific subset of kinase genes (e.g., *OsCIPK2* and *OsCIPK5*) under PEG stress leads us to propose a putative “partner switching” hypothesis. Rather than functioning solely within the SOS pathway under salinity, OsCBL4 might possess the plasticity to associate with distinct CIPK partners in a context-dependent manner. Under direct osmotic stress, the dramatic up-regulation of *OsCIPK2* mirrors the osmotic induction of *OsCBL4*, suggesting a coordinated transcriptional preparation that could facilitate their selective interaction for osmotic adjustment.

While these promoter and expression profiles offer valuable insights into the potential drought-responsive roles of the *OsCBL-OsCIPK* network, the central mechanistic claims require further substantiation. To clarify the precise nature of the *OsCBL4*-mediated osmotic response and to definitively test the proposed partner switching and interaction selectivities, rigorous in vivo functional assays—including mutant phenotyping and localized expression studies under whole-plant water-deficit conditions—are necessary.

### 4.3. Co-Regulation Mechanism and Interaction Specificity in the OsCBL-OsCIPK Network

One of the most scientifically significant insights from our study lies in the striking “Expression–Interaction Coherence” revealed within the rice calcium signaling network. Our data uncover a sophisticated co-regulatory mechanism in which the physical interaction capacity of the calcium sensor *OsCBL4* is tightly coordinated with the transcriptional activation of its downstream kinase partners under stress conditions. Such coordinated regulation between gene expression and protein interaction has been increasingly recognized as a central principle in plant stress signaling networks, ensuring both specificity and efficiency of signal transduction [13].

The integration of transcriptional profiling and protein interaction data suggests a degree of coherence in the rice CBL-CIPK network under osmotic stress. Our Y2H assays indicate that OsCBL4 can interact with a subset of kinases, including OsCIPK2, OsCIPK5, OsCIPK9, and OsCIPK18, which also exhibit transcriptional up-regulation under PEG-simulated drought. For example, *OsCIPK2*, showing dramatic upregulation under PEG treatment, displays robust binding affinity with OsCBL4. This pattern hints at a potential co-regulatory mechanism where transcriptional induction and physical interaction capabilities might be functionally coupled to modulate signaling during environmental stress [13,35].

Conversely, the lack of interaction between OsCBL4 and OsCIPK30 in our Y2H system aligns with the observation that *OsCIPK30* transcript levels were relatively unresponsive to the tested stress treatments. While this suggests a level of interaction specificity that could prevent non-productive protein complex formation under stress, it is important to note that these interactions require further in vivo validation under physiological stress conditions to confirm their biological relevance [35].

This concept of a “Dual-Lock Mechanism” requiring both transcriptional activation and specific protein interactions for signal propagation, offers a plausible model for how plants might optimize stress responses [36,37].

A notable observation from our study is the coordinated expression and interaction of the OsCBL4–OsCIPK9 pair. Both genes were induced by PEG but showed limited responsiveness to exogenous ABA under our specific assay conditions. While this could imply an osmotic-specific, ABA-independent regulatory module, this interpretation must be approached with caution. The apparent lack of ABA response might be influenced by factors such as treatment dosage, timing (stress window effects), or tissue-specific sensitivities that were not fully captured in our current experimental setup. Further whole-plant functional studies and detailed spatio-temporal expression analyses are necessary to determine whether this module truly operates independently of the canonical ABA pathway. If confirmed, such modules could provide valuable targets for understanding plant stress adaptation and potentially informing future crop improvement strategies, though direct evidence linking this specific interaction to whole-plant drought tolerance and yield maintenance remains to be established.

## 5. Conclusions

This study provides a comparative genomic and functional survey of the CBL and CIPK families across five grass species. Phylogenetic and synteny analyses indicate that these families expanded primarily through segmental duplication events. In rice, promoter architecture and expression profiling identify *OsCBL4* and a specific subset of *OsCIPKs*, such as *OsCIPK2* and *OsCIPK9*, as key components of the osmotic stress response. Notably, *OsCBL4* exhibits a distinct transcriptional induction under PEG-simulated drought that appears separate from canonical ABA-dependent regulation under the tested conditions. Physical interaction assays, including Y2H and LCI, demonstrate that OsCBL4 selectively recruits these drought-responsive kinases. These findings suggest a co-regulation mechanism where transcriptional induction and protein interaction specificity coordinate to mediate osmotic signaling. While this work establishes a structural and transcriptional framework for the OsCBL-OsCIPK network, further in vivo studies using genetic mutants are necessary to validate the specific roles of these modules in whole-plant drought tolerance.

## Data Availability

All data generated or analyzed during this study are included in this published article. Further inquiries can be directed to the corresponding authors.

## Supplementary Materials

The following supporting information can be downloaded at: https://www.mdpi.com/article/10.3390/genes17030345/s1, Figure S1: Comparative phylogenetic analyses of the CBL and CIPK gene families in Five Grass Species. Figure S2: Subcellular localization predictive analysis of OsCBL and OsCIPK proteins families. Figure S3: Expression patterns of OsCBL and OsCIPK families under pathogen (P6) and MeJA stresses. Figure S4: Yeast self-activation verification showed that OsCIPK2/5/8/9/11/18/30 had no self-activation activity. Table S1: Primer sequences used in this study for qRT-PCR. Table S2: Primer sequences used in this study for vector construction.
