# Supplementary material for: Evolutionary Dynamics of the CBL-CIPK Gene Families in Five Grasses and Expression/Interaction Analysis in Rice: Focus on an OsCBL4-Associated Module
Source: Genes (Basel). 2026 Mar 19;17(3):345. doi: 10.3390/genes17030345 (PMC13026453; doi:10.3390/genes17030345)
Supplement: Supplementary file 1 [file genes-17-00345-s001.zip › genes-4200783-supplementary.pdf]

A

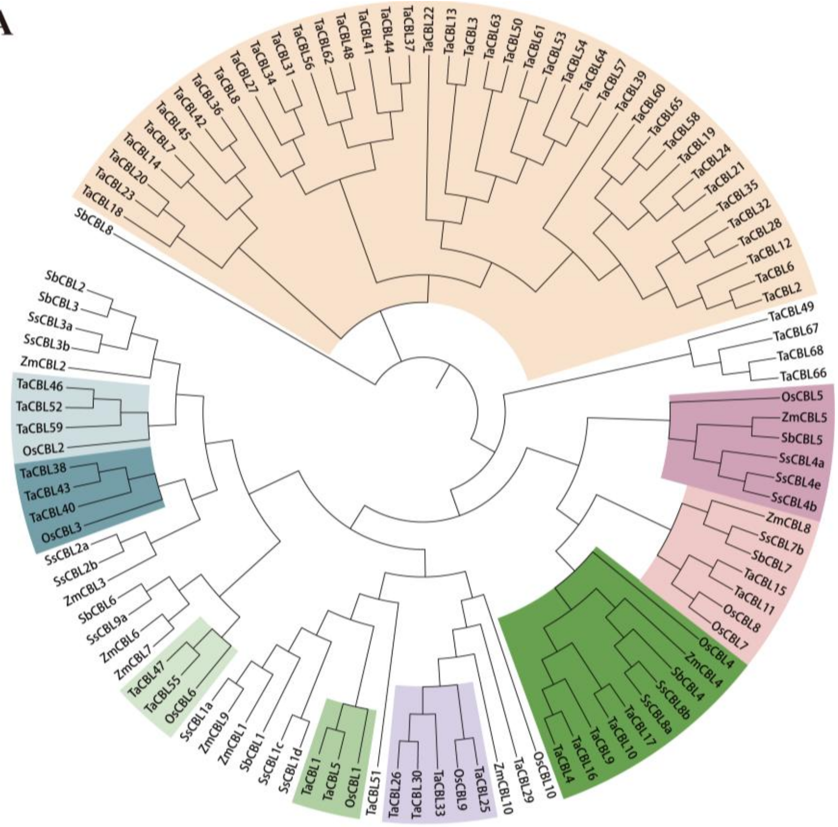

B

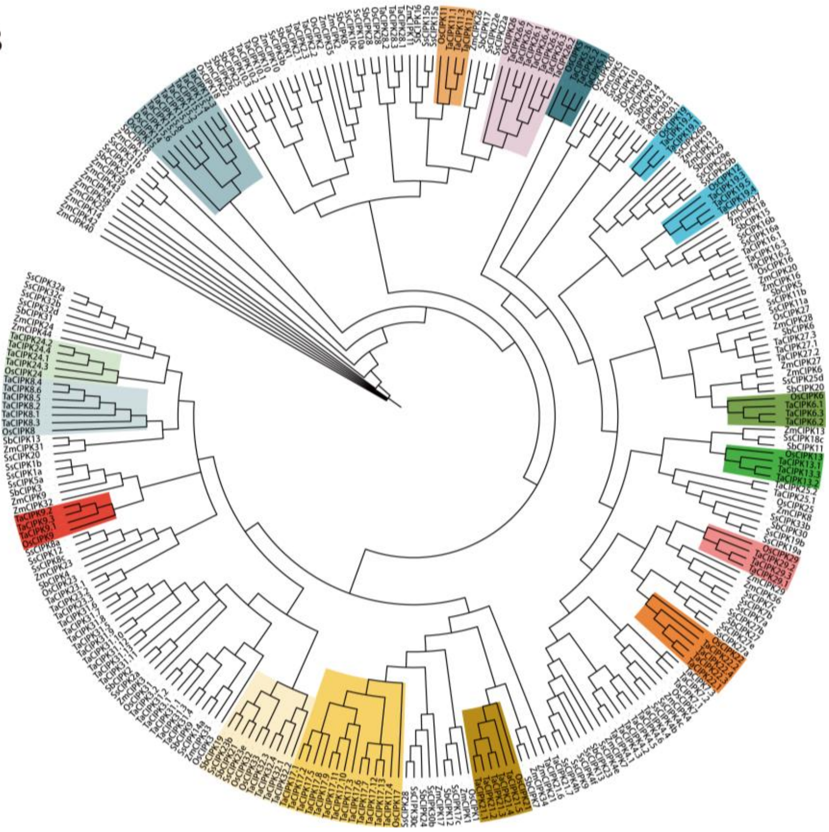

Figure S1. Comparative phylogenetic analyses of the *CBL* and *CIPK* gene families in Five Grass Species. A. Comparative phylogenetic analyses of the *CBL* gene families in Five Grass Species. B. Comparative phylogenetic analyses of the and *CIPK* gene families in Five Grass Species.

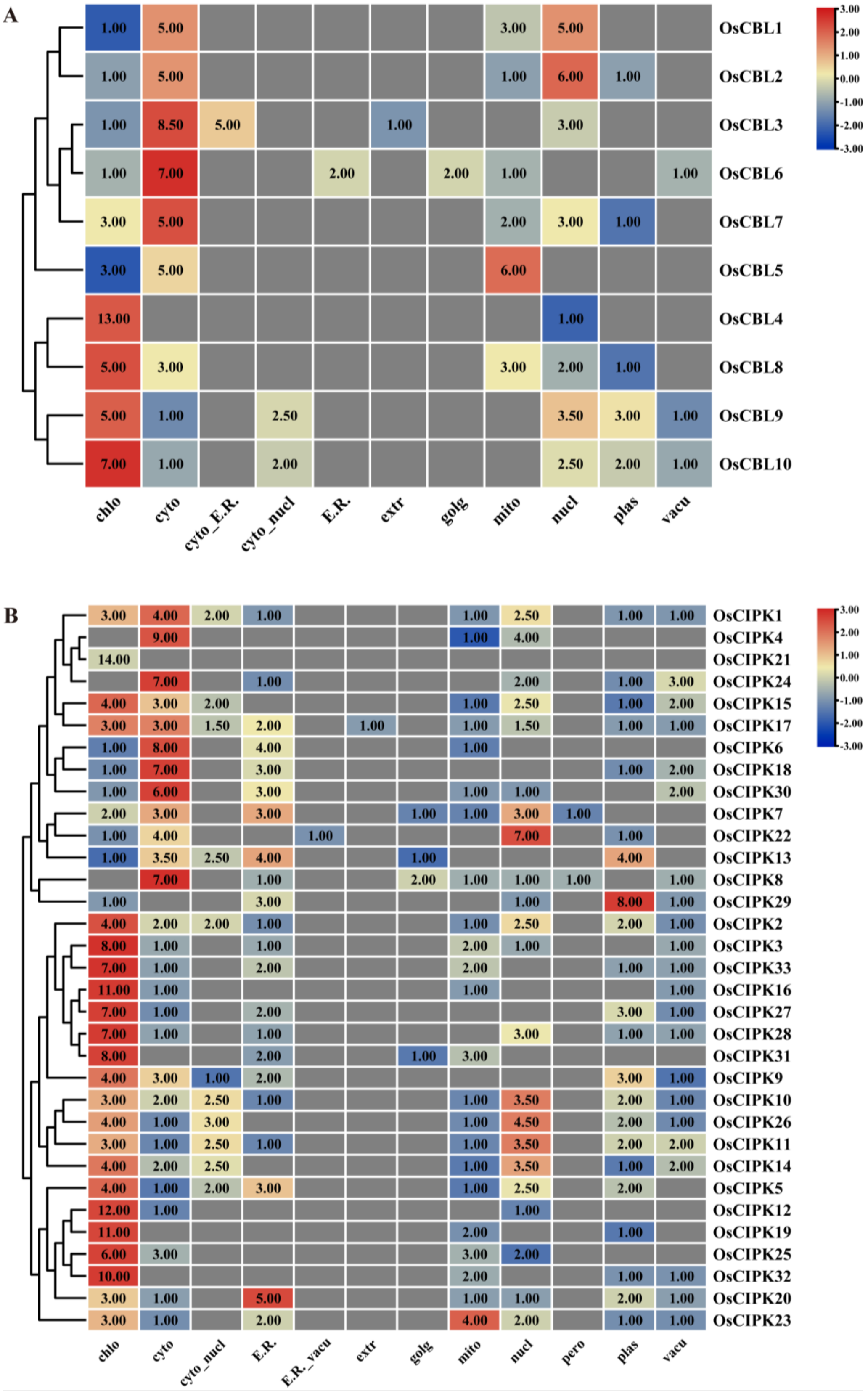

Figure S2. Subcellular localization predictive analysis of OsCBL and OsCIPK proteins families. A. **Os**CBL proteins families. B. **Os**CIPK proteins families. Values in squares indicates the predicted score of subcellular localization. The legend on the right shows scores and corresponding colors from TBtools analysis, reflecting row data in the matrix after Row Scale. In single-row data, a deeper red indicates more components, while a blue hue signifies fewer. Grey blocks denote absence of components.

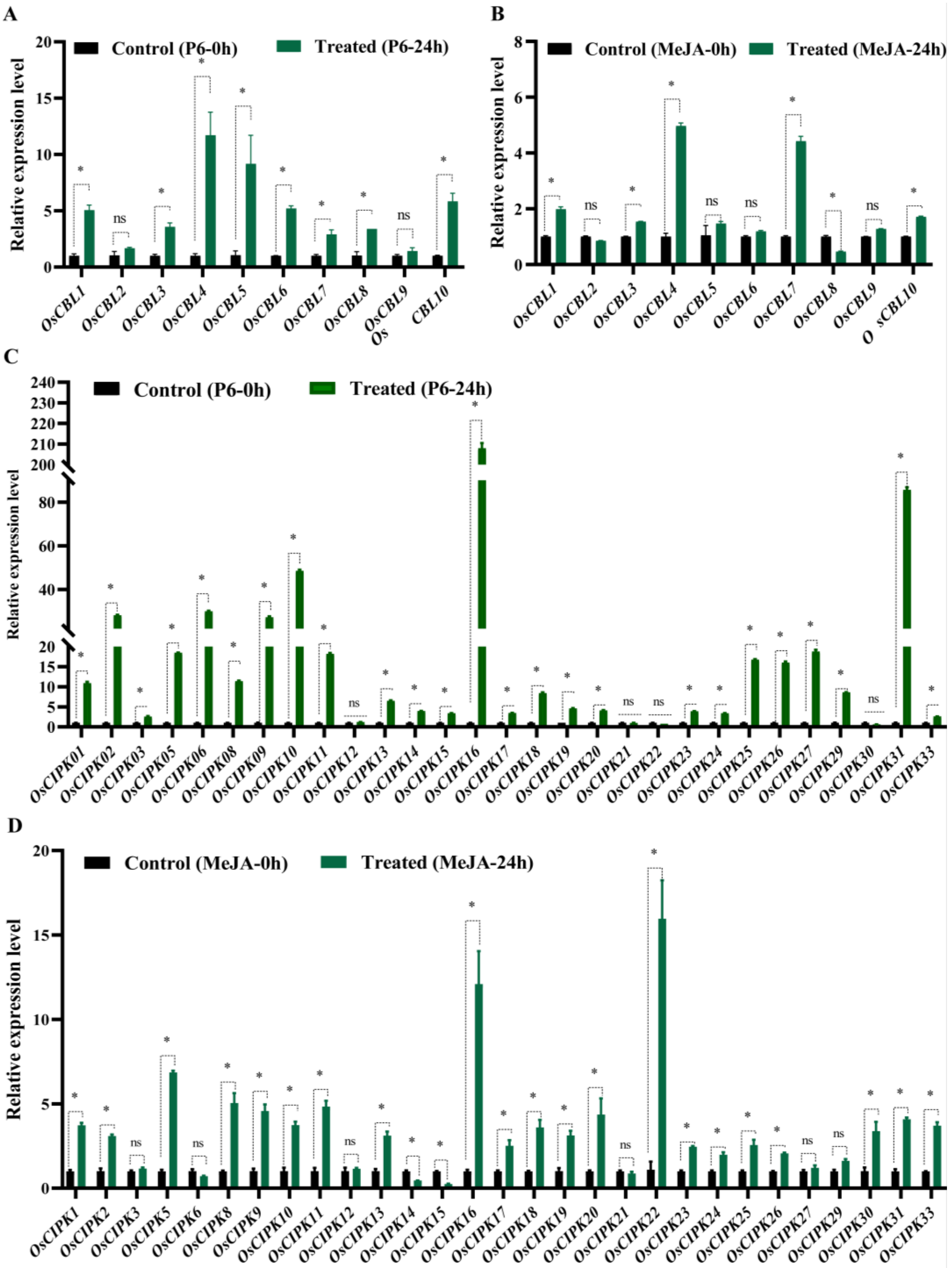

Figure S3. Expression patterns of *OsCBL* and *OsCIPK* families under **pathogen (P6)** and MeJA stresses. A. Expression patterns of *OsCBL* gene families under **pathogen (P6)** stress. **B.** Expression patterns of *OsCBL* gene families under MeJA stress. C. Expression patterns of *OsCIPK* gene families under **pathogen (P6)** stress. D. Expression patterns of *OsCIPK* gene families under MeJA stress.

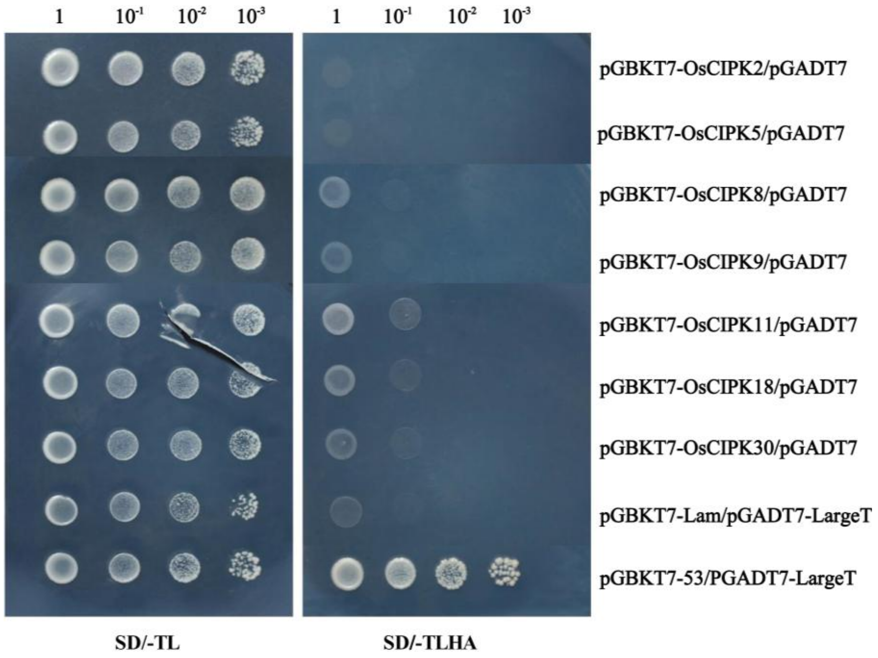

Figure S4. Yeast self-activation verification showed that OsCIPK2/5/8/9/11/18/30 had no self-activation activity.

Table S1. Primer sequences used in this study for qRT-PCR.

Primer Name	Forward primer Sequence (5′->3′)	Reverse primer Sequence (5′->3′)
OsCBL1	TTCTGTGATCGACGATGGGC	TCACCAAAGTCGATGACCCC
OsCBL2	GCGCAATCTGGTTCTTCGTC	TGTCATCGACCTGGGAATGG
OsCBL3	GCTACTCAAGTGCTGCGACC	CATACAACGCCTCAACCTCG
OsCBL4	CGCCCAAACCACCTTTACG	AGGCAAGCTGAAACTCCTCC
OsCBL5	GATGGTGTTGGCTCTTCTGGA	TCTCTCATCGCCTTTTGTGTCA
OsCBL6	AGCCTTGACAGCTCCAATCG	AACAGCTCATACAGCGCCTC
OsCBL7	CGCTGTTGAGCAGATTGTGG	CTTGCGAACGCCTTCCATTC
OsCBL8	GCACGAAATGGTGCTTGCTC	GTCTGCTTGCTTGAACGTCTG
OsCBL9	GGAATTCGTATTGCGGCATCC	AACCGCGGTGTTGAAGACAA
OsCBL10	GAACACCGGCTGGAAAGAATC	CACTTATTGCGTGAATAAACTCTTC
OsCIPK1	CCACAGAGACCTTAAGCCCG	GCCATCGTAACCTCGGTTCT
OsCIPK2	GAGCACGTCAAAGGTGGAGA	CGGTGATAAACACCCCTGCT
OsCIPK3	AGCAACTGATCAACGCCGTA	ACTTTGATGTTCCCGGCAGT
OsCIPK5	ATTGCGTGTCGGCATGATTG	CTGGCTACCCTGCTGAAGAG
OsCIPK6	TCAAGAAGACGTCCATCTCCAG	CCTTCTCGGCCTCCTCCTTAG
OsCIPK8	GTTGCCCCAGAGGTTCTCAG	GGCACCATTAGGAAACCAAGC
OsCIPK9	TTTGGCCTTAGTGCGTTTGC	GGTTGGGGTCATCGAAAGGT
OsCIPK10	GGTCGTGCTAACTCCCACAC	CTCACTGAATCTCCCTGCGT
OsCIPK11	GGCCGTTATGAGGTTGGGAA	CAACAGCTTGGCCGGTAGTA
OsCIPK12	TAGGATCACCGTGCCAGAGA	GGATCAGCAGGCTCCAAGTT
OsCIPK13	AACTTCGCCAAGGTGTACCA	GTGAGCCCCGACTTGAAGAT
OsCIPK14	GCCTGAAAACCTGCTGTTGG	GCCATCTTGCCTCTTCGACT
OsCIPK15	AGATGATGGACAAACAGCAGATA	GTTCTTATGAGCCACCAACCG
OsCIPK16	TTCCAGCACGAGAACTACGC	CCTTCTTGAACCATGGCGTG
OsCIPK17	ATTGAAAGCTCAGCGACGGA	CAGGGTCTCCGTTGGACTTC
OsCIPK18	ATCGCGATCTGAAGCCAGAG	CCGTCATACCCTTTTCGGCT
OsCIPK19	AGACCTCTCGGGTCTCTCAC	TCCCTCGCTCCTCAAACAAC
OsCIPK20	ACCAATGGCGAGTTCAAGGT	TAGGGTTTGGGTCGAGGAGT
OsCIPK21	AGTATGAATCCGGTCACCGAC	TGGGAATTCGGCTGGGTTTT

OsCIPK22	ATGATCCAAGCTGCGTCCTC	CTGACTTGGAATCCCCACCC
OsCIPK23	AAGCAATCGCAGGGTTCTGT	TTAAAGCCCATGGGTCCAGC
OsCIPK24	CAGAATGAGCCCGTTTGCTG	GTGTCACCAGCAACCTTTCG
OsCIPK25	CCGACATTATCGCCAAGCTG	CACACCTGGACCATGACGAG
OsCIPK26	AACCGGCGGCAGCTATATTT	GCTACCAACCTCAGAACCCC
OsCIPK27	CGGCAACTTCGGTCGTG	CTTGTCCTTGGACACCACCTTC
OsCIPK29	GAGAAGGGAAGAAGGGCACC	GCGATCTTGAACACGCAGAC
OsCIPK30	CTGCTTGCCAGAAAGGAAGC	GACCGCGAGATGATGTCGAA
OsCIPK31	TGGTTGAACAGATTAGGCGTGA	TTCCCATGACCTCGAACAGC
OsCIPK33	AGATCTGCTCCCCTTGCCTA	GGGTGCAACCTGCAGTATCT
OsUBQ10	TGGTCAGTAATCAGCCAGTTTGG	GCACCACAAATACTTGACGAACAG

Table S2. Primer sequences used in this study for vector construction.

Primer Name	Forward primer Sequence (5′->3′)	Reverse primer Sequence (5′->3′)
OsCBL4-pGADT7	ccagattacgctcatatgATGGGATGCGCGTCGT	ctcgagctcgatggatccTCAG-TCATGGGCTTCTGAATGC
OsCIPK2-pGBKT7	gaggaggacctgcatatgATGGCGGAGCAGAGAGGA	ctgcaggtcgacggatccTTAGCACGTT-GGCTGCTGC
OsCIPK5-pGBKT7	gaggaggacctgcatatgATGGAGAA-GAAGGCGTCCATCC	ctgcaggtcgacggatccTTAAATGGCATGTCTCGA-GATTGACTT
OsCIPK8-pGBKT7	gaggaggacctgcatatgATGGTGGGCGGAGGG	ctgcaggtcgacggatccCTAGCGCTTCGA-TAGCCGC
OsCIPK9-pGBKT7	gaggaggacctgcatatgATGGCGGAGGCGGAGG	ctgcaggtcgacggatccTCACCTCTTCTTT-GCTGCTTTTGC
OsCIPK11-pGBKT7	gaggaggacctgcatatgATGATGGATGG-GAGGTCAATCTTGA	ctgcaggtcgacggatccCTAATTCTTTGTTT-GTTCTGCGGGGATA
OsCIPK18-pGBKT7	gaggaggacctgcatatgATGATGGAACTTGAGAA-GAATGGAAACAT	ctgcaggtcgac-ggatccCTATGGCTGGCCATGATCTTCAG
OsCIPK30-pGBKT7	gaggaggacctgcatatgATGGCCATGGAGACGACGA	ctgcaggtcgacggatccTCAA-GAGCGCTTCGTGATCG
OsCBL4-nLUC	cgggggacgagctcggtaccATGGGATGCGCGTCGT	gcgtacgagatctggtcgac-GTCATGGGCTTCTGAATGC
OsCIPK9-cLUC	acgcgtcccggggcggtaccATGGCGGAGGCGGAGG	gtcgacctgcagagctttcgTCACCTCTTCTTT-GCTGCTTTTGC
